# Plasmonic Absorption Enhancement in Elliptical Graphene Arrays

**DOI:** 10.3390/nano8030175

**Published:** 2018-03-19

**Authors:** Jiajia Chen, Yu Zeng, Xibin Xu, Xifang Chen, Zigang Zhou, Pengcheng Shi, Zao Yi, Xin Ye, Shuyuan Xiao, Yougen Yi

**Affiliations:** 1 School of Science, Southwest University of Science and Technology, Mianyang 621010, China; chenjiajia522@outlook.com (J.C.); yuzeng7@hotmail.com (Y.Z.); chenxifang1987@swust.edu.cn (X.C.); zhouzigang@swust.edu.cn (Z.Z.); shi623672967@outlook.com (P.S.); 2 Joint Laboratory for Extreme Conditions Matter Properties, Southwest University of Science and Technology, Mianyang 621010, China; 3 Research Center of Laser Fusion, China Academy of Engineering Physics, Mianyang 621900, China; dodolong@csu.edu.cn; 4 Wuhan National Laboratory for Optoelectronics, Huazhong University of Science and Technology, Wuhan 430074, China; 5 College of Physics and Electronics, Central South University, Changsha 410083, China; yougenyi@csu.edu.cn

**Keywords:** graphene surface plasmon, metamaterials, absorption enhancement, FDTD method

## Abstract

In this paper, we come up with a wavelength tunable absorber which is made up of periodically elliptical graphene arrays in the far-infrared and terahertz regions. Through simulation, we find that we can increase the length of long axis of the ellipse, raise the incidence angles of TM- and TE-polarization (TM- and TE-polarization indicate the direction of the incident electric field along the direction of the x and the y axis, respectively.) within certain limits, and increase the chemical potential of graphene, so as to enhance the absorption of light in the elliptical graphene arrays. We also compare the absorption spectra of the original structure and the complementary structure, and find that the absorption of the original structure is higher than that of the complementary structure. In the end, we study the changes in the absorption rate of the double layer structure of the elliptical array with the increase in the thickness of SiO_2_. The elliptical array structure can be applied to tunable spectral detectors, filters and sensors at far-infrared and terahertz wavelengths.

## 1. Introduction

Since the first introduction of its synthesis via a ‘Scotch tape’ method in 2004, graphene has aroused great interest in the scientific community [1]. As a single-layer carbon atom arranged in a two-dimensional lattice, graphene shows unique and excellent mechanical [2,3], thermal [4] and electromagnrtic properties [5,6,7,8].

Although monolayer graphene absorbs only 2.3% of the incident energy in the visible and near-infrared ranges, in the mid-infrared and terahertz ranges, graphene is able to concentrate very large amounts of light energy. This is precisely because graphene supports the surface plasmon resonance (SPR) like metals. The graphene, which supports surface plasmon resonance, has many excellent properties that can supplement the defects of the metal. This is because the graphene, which supports SPR, is beneficial to the absorption of light energy [9]. Recently, a large number of patterned graphene arrays have been studied, and it has been proved that they can enhance the absorption of light energy by making use of the SPR of graphene by means such as strips [10,11], rings [12,13], crosses [14,15,16] and so on. However, there has been less research conducted on elliptical graphene arrays. 

In this work, we propose the study of optical absorption enhancement based on elliptical graphene. On the basis of silicon, we add a layer of silica with elliptical graphene. Due to the unique asymmetry of the ellipse, the proposed model possesses the advantage of having more free geometric parameters compared with the above-mentioned symmetric structures, and offers good flexibility for the manipulation of light. We not only discuss the influence of structural parameters and chemical potential, but also discuss the complementary structure and the double layer structure. We also study the changes in the resonant wavelength and absorption peak caused by different directions of the incidence of light in the polarization of TE and TM. Thus, the elliptical array structure can be applied to tunable spectral detectors, filters and sensors at far-infrared and terahertz wavelengths.

## 2. Geometry of the Elliptical Graphene Array

**Figure 1:**
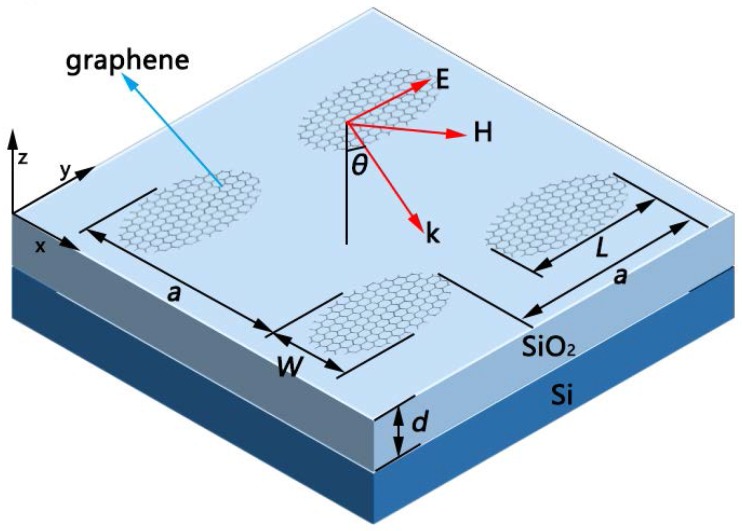
Geometric schematic diagram of the elliptical arrays of graphene with the short axis *W*, the major axis *L* and period *a*. The arrays have a Si substrate covered with a SiO_2_ layer with thickness d. The incident angle is θ.

The geometric schematic diagram of our elliptical graphene array is shown in Figure 1. The structure consists of an elliptical array of graphene with the major axis L, the short axis W, and period a. The silicon dioxide structure, close to the graphene array, has the thickness d. The bottom is silicon. The refractive index of silicon is *n*_si_ = 3.4, and the relative permittivity of silicon dioxide is *ε*_d_ = 3.9 [16,17,18]. Here, a plane wave with incident angle θ is used to illuminate this system, and the *x*-*z* plane is taken as the plane of incident. The Kubo formula, consisting of interband and intraband transitions, provides the surface conductivity of graphene σ [19,20]. In the THz and far-infrared regime, on account of *μ*_c_ >> *K*_B_*T*_m_, the interband contribution can accordingly be neglected because of the Pauli exclusion principle. Thus, the conductivity of graphene is close to the Drude-like model

(1) $$\sigma (\omega )\;=\;\frac{e^{2}\mu_{c}}{\pi {\bar{h}}^{2}}\;\frac{i}{\omega \;+\;i/\tau }$$

where e is the electron charge and $\bar{h}$ is the reduced Plank’s constant [21]. Equation (1) depends on the chemical potential *μ*_c_, relaxation time τ and photon frequency *ω*. 

In this work, we first set the chemical potential and relaxation time to *μ*_c_ = 0.4 eV and *τ* = 0.5 ps, respectively. The influence of chemical potential will be analyzed later. Through the finite-difference time-domain (FDTD Solutions, Lumerical Inc., Vancouver, BC, Canada) method, we simulated the model and calculated the absorption performance. In the x and y directions, we used symmetric and anti-symmetric boundary conditions, respectively. In the propagation of the incident plane waves, perfectly matched layers are utilized in the *z* direction. The reflection, transmission, and absorption can be expressed as follows:

(2) $$R(\omega )\;=\;|S_{11}(\omega {)\;|}^{2}$$

(3) $$T(\omega )\;=\;|S_{21}(\omega {)\;|}^{2}$$

(4) A(ω)=1−T(ω)−R(ω)

which are obtained by the *S*-parameters. 

## 3. Simulation Results and Discussions

**Figure 2:**
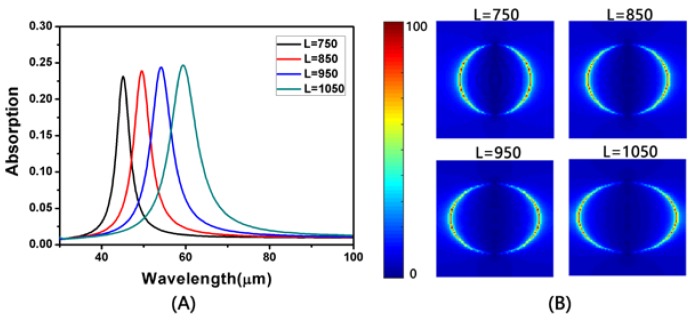
(**A**) The absorption spectrum of the long axis with different lengths at *W* = 750 nm; (**B**) The distributions of electric field (|*E*|) at absorption peaks of *L* = 750 nm, 850 nm, 950 nm and 1050 nm. *a* = 2.5 μm and *d* = 0.3 μm.

**Figure 3:**
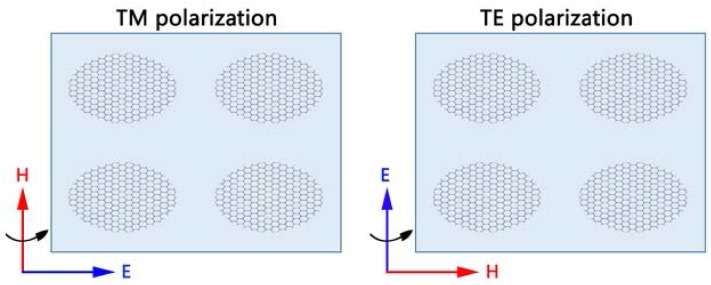
TM- and TE-polarization schematic diagram.

At first, we studied the geometrical effects of the absorption of monolayer elliptical graphene array at normal incidence. When the long axis is changed and the short axis is constant, we can see clearly from Figure 2A that there are obvious absorption peaks in both the far-infrared and the terahertz bands. In Figure 2A, the result of fixing the short axis to 750 nm shows that the wave peak is constantly rising and moving toward the long wave as the long axis increases. The peak value rises from about 23% to 25%, and the resonance wavelength also moves from about 45 to 59 μm. It is not difficult to understand that the enhancement of absorption should be attributed to the SPR of graphene. Figure 2B shows an electric field map corresponding to the long axis of different lengths to help us better understand the reasons for the absorption enhancement. When the long axis increases, the gap between the adjacent elliptical graphene arrays in the long axis decreases, resulting in an increase in the coupling between them. The increase of coupling leads to the red shift and enhancement of the absorption peak. From the electric field distribution diagram, a local intensification of the electromagnetic field, which is due to the strong electric dipole resonance excited by the charge of the two ends of the long axis, can be achieved. This strong resonance can effectively catch the energy of light and make enough time to eliminate the ohm loss in graphene. As shown Figure 3, the TM-polarization indicates that the direction of the incident electric field is along the x axis. The TE-polarization indicates that the direction of the incident electric field is along the y axis.

**Figure 4:**
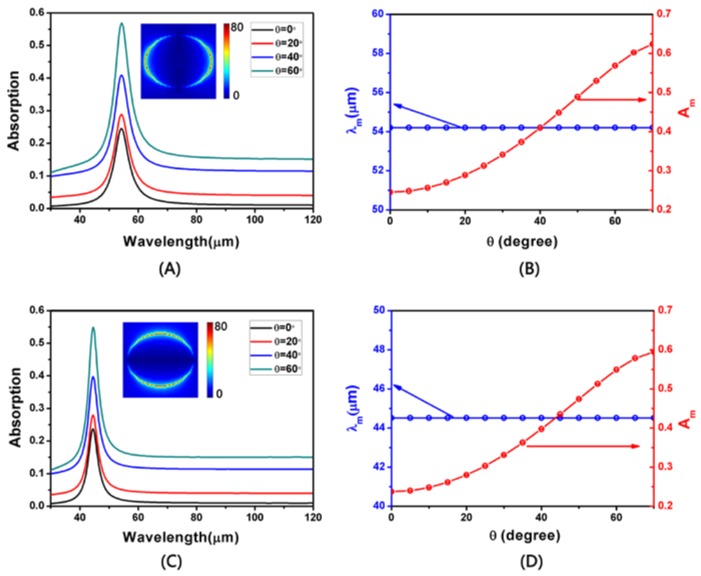
(**A**,**C**) The simulated absorption at different incident angles on TM- and TE-polarization, respectively. The inserts indicate the distributions of electric field (|*E*|) at θ = 0°. (**B**,**D**) The resonance wavelength and absorption maximum at different incident angle on TM- and TE-polarization, respectively.

At *L* = 950 nm, *a* = 2500 nm, *d* = 300 nm and *W* = 750 nm, the absorption spectra for TM- and TE-polarization are displayed in Figure 4 for different incidence angles. It is obvious that the resonance wavelength is not affected by the change of the incidence angle. On the contrary, the absorption peak at the resonant wavelength is more sensitive to the change of the incidence angle. For the polarization of TM and TE, the resonance wavelengths are about 54 and 44 μm, respectively, and the maximum absorption value increases with an increase of the incidence angle. By comparison, we can see that the resonance wavelength of the TE polarization is blue shifted compared to that of TM, and the width of the resonance wavelength of the TE polarization is narrower. The difference in resonance wavelengths of these two modes is due to the difference in the values of the long and short axes of the elliptical graphene array in the directions of *x* and *y*. Then we can clearly see that regardless of the mode, when the angle is between a minimum of 0° and a maximum of 70°, the absorption is increased by about 32%, which is two times greater than the original one. The cause of this phenomenon is that, as the incidence angle increases, the reflection of light becomes weaker. In Figure 4A,C the inserts show the electric field distribution at the absorption peak of θ = 0° for TM- and TE-polarization, respectively.

**Figure 5:**
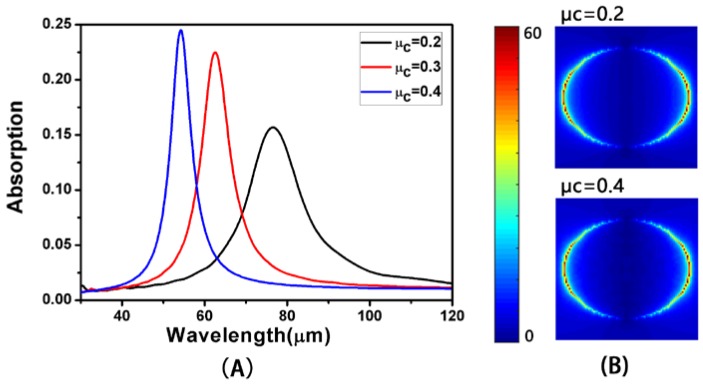
(**A**) The simulated absorption with different chemical potentials; (**B**) The distributions of electric field (|*E*|) at the absorption peak for *μ*_c_ = 0.2 and 0.4 eV.

The chemical potential μ_c_ plays a decisive role in the optical properties of graphene [22,23]. At *L* = 950 nm, *a* = 2500 nm, *d* = 300 nm and *W* = 750 nm, the absorption spectra at normal incidence are displayed in Figure 5A for different values of μ_c_. The results show that the chemical potential of graphene increases the blue shift of the spectrum, the resonance wavelength is shortened, and the absorption rate is increased. This is because when the chemical potential of graphene increases, the wavelength of the plasmons, such as the corresponding graphene, will increase. At the same time, with the increase of chemical potential, the conductivity of the surface of the graphene will increase as well. Therefore, the absorption of light is enhanced [13,22]. This is why the graphene layer absorbs more light when the chemical potential of graphene is increased. Figure 5B reveals the electric field distributions at the absorption peak for *μ*_c_ = 0.2 and 0.4 eV. With the increase in the chemical potential of graphene, the SPR of graphene is enhanced. The enhancement of the local electric field leads to an increase in absorption. Therefore, we are able to control the chemical potential of graphene in order to achieve control of the absorption. 

**Figure 6:**
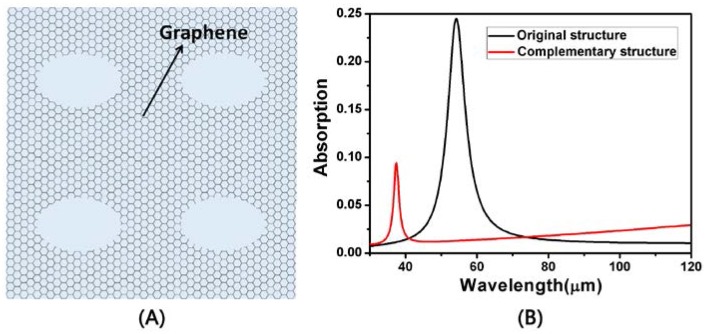
(**A**) The top view of the complementary structure of Figure 1; (**B**) Absorption spectra of the original structure and the complementary structure under the same conditions.

We also studied the absorption spectra of the complementary structure under the same conditions. Figure 6A shows the top view of the complementary structure with the same structure. With *L* = 950 nm, *a* = 2500 nm, *d* = 300 nm and *W* = 750 nm, Figure 6B shows the absorption spectrum of the two structures. We can clearly see that the resonant wavelength of the complementary structure is blue shifted compared to the original structure, and the absorption peak of the complementary structure decreases. 

**Figure 7:**
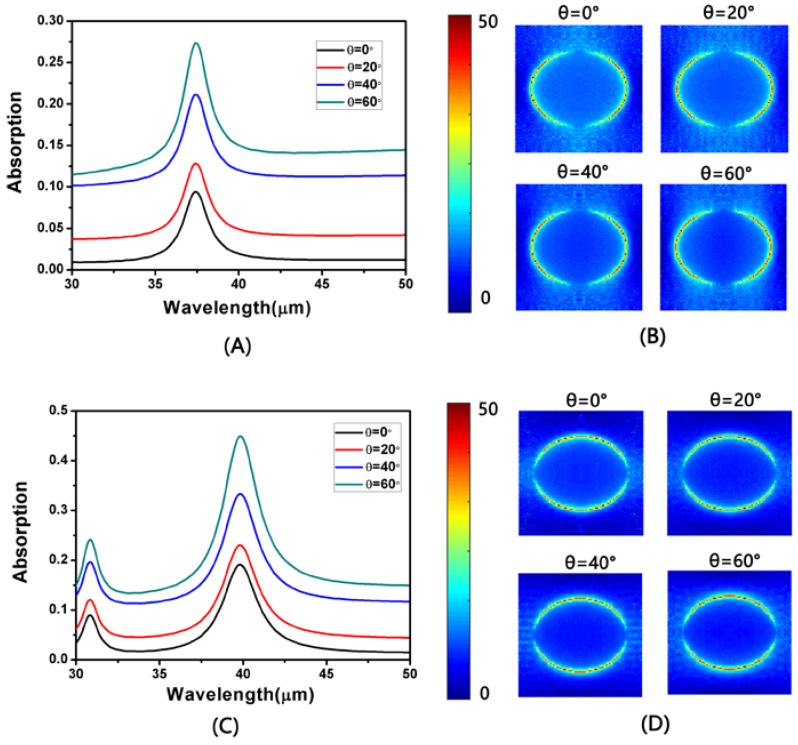
(**A**,**C**) The simulated absorption at different incidence angles for TM- and TE-polarization, respectively; (**B**,**D**) The distributions of the electric field (|*E*|) at the absorption peak for different incidence angles for TM- and TE-polarization, respectively.

For the complementary structure, we also studied the absorption of the TM and TE modes at different incidence angles. With *L* = 950 nm, *a* = 2500 nm, *d* = 300 nm and *W* = 750 nm, the absorption spectra for TM- and TE-polarization are displayed in Figure 7A,B for different incidence angles. As can be seen from the diagram, the absorption peak of the resonant wavelength of the TM and TE modes increased with the increase in the incidence angle. Figure 6B,D shows the electric field distribution at the absorption peak for TM- and TE-polarization, respectively. From the electric field distribution diagram, it can be seen that the stronger local electric field will lead to greater light absorption of graphene. 

**Figure 8:**
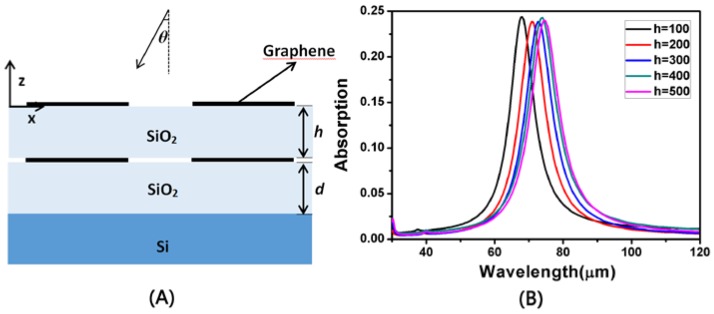
(**A**) The side view of the double-layer structure of Figure 1; (**B**) Absorption spectra of the original structure and the complementary structure under the same conditions.

Finally, we studied the double layer structure of the elliptical graphene array. With *L* = 950 nm, *a* = 2500 nm, *d* = 300 nm and *W* = 750 nm, Figure 8B shows the change of absorptivity of the double layer structure with the thickness *h* of SiO_2_. It is obvious that when *h* is increased, the resonance wavelength undergoes a red shift, and the absorption peak at the resonance wavelength moves slightly up and down. This is due to the change in the distance between the two graphene arrays, which results in a change in the coupling between them.

## 4. Discussion

To conclude, we propose a wavelength-tunable absorber that is made up of periodic elliptical graphene arrays. By increasing the length of the major axis of the ellipse, increasing the incidence of x polarization within a certain range of angles, or increasing the graphene chemical potential, we can achieve tuning of the graphene resonant wavelength and raise the absorption rate of graphene. Among these adjustments, the change of the angle of incident light can increase the absorption rate to 56%. This is attributed to the SPR of graphene. We also found that the absorption of the structure is greater than that of its complementary structure. We compare the absorption changes caused by the incidence angle changes in the TM and TE modes. A larger angle of incidence causes higher absorption. In the end, we study the increase in the absorption of the double layer structure of the elliptical array with the increase in the thickness h of SiO_2_. Thus, the elliptical array structure we propose can be applied to tunable spectral detectors, filters and sensors at far-infrared and terahertz wavelengths.
